# PD-L1(+) tumor-associated macrophages induce CD8(+) T Cell exhaustion in hepatocellular carcinoma

**DOI:** 10.1016/j.neo.2025.101234

**Published:** 2025-09-29

**Authors:** Takuto Nosaka, Masahiro Ohtani, Junki Yamashita, Yosuke Murata, Yu Akazawa, Tomoko Tanaka, Kazuto Takahashi, Tatsushi Naito, Yoshiaki Imamura, Kenji Koneri, Takanori Goi, Yasunari Nakamoto

**Affiliations:** a Second Department of Internal Medicine, Faculty of Medical Sciences, University of Fukui, Fukui, 910-1193, Japan; b Division of Diagnostic Pathology/Surgical Pathology, University of Fukui Hospital, Fukui, 910-1193, Japan; c First Department of Surgery, Faculty of Medical Sciences, University of Fukui, Fukui, 910-1193, Japan

**Keywords:** Hepatocellular carcinoma, Tumor-associated macrophages, PD-L1, CD8, Tumor immune microenvironment

## Abstract

The therapeutic efficacy of immune checkpoint inhibitors (ICIs) in patients with hepatocellular carcinoma (HCC) is profoundly influenced by the tumor immune microenvironment (TIME), where tumor-associated macrophages (TAMs) expressing programmed death-ligand 1 (PD-L1) serve as key modulators of immune suppression and tumor progression. Although PD-L1(+) TAMs have attracted increasing attention, their precise immunological functions in patients with HCC remain incompletely understood. In this study, we conducted an integrated analysis combining single-cell transcriptomics, spatial profiling, *in vitro* functional assays, and *in vivo* therapeutic modeling to clarify the role of PD-L1(+) TAMs in HCC. Single-cell RNA sequencing of tumor samples from patients with HCC (GSE189903) revealed that intratumoral PD-L1(+) TAMs were enriched for immune-related signaling pathways and expressed chemokines including CXCL9, CXCL10, and CXCL11. In vitro, GM-CSF–induced PD-L1(+) macrophages promoted CD8(+) T cell exhaustion, characterized by increased expression of TIM3 and suppression of cytotoxic molecules such as GZMB. Spatial analysis using multiplex immunofluorescence staining of surgical specimens from 113 patients with HCC demonstrated that close proximity between PD-L1(+) TAMs and CD8(+) T cells within tumors was an independent predictor of early postoperative recurrence and poor outcome. Moreover, in a murine orthotopic liver cancer model, the combination of anti–GM-CSF and anti–PD-L1 antibodies inhibited the differentiation of PD-L1(+) TAMs, reduced their contact with CD8(+) T cells, alleviated T cell exhaustion, and potentiated antitumor immunity. These findings highlight the critical contribution of PD-L1(+) TAMs to immune evasion in patients with HCC and support their therapeutic targeting as a strategy to enhance ICI responses.

## Highlights

• PD-L1(+) TAMs exhibit immune-regulatory chemokine expression in HCC.
• GM-CSF–induced PD-L1(+) macrophages promote CD8(+) T cell exhaustion *in vitro*.
• Proximity between PD-L1(+) TAMs and CD8(+) T cells indicates poor prognosis in HCC.
• Inhibiting PD-L1(+) TAMs restores CD8(+) T cell–mediated antitumor immunity.

## Introduction

Liver cancer is the sixth most common neoplasm and the third leading cause of cancer-related death globally, with hepatocellular carcinoma (HCC) constituting approximately 85–90% [1]. With advancements in systemic therapy for advanced HCC, immune checkpoint inhibitor (ICI)-based treatments have emerged as pivotal components of systemic first-line therapy [2]. However, only a few patients respond to ICI-based therapy. The efficacy of ICI-based therapy was largely related to the characteristics of the tumor immune microenvironment (TIME) [2,3].

Tumor-associated macrophages (TAMs) are key in promoting cancer progression by influencing tumor development, proliferation, invasion, or treatment response [4]. Macrophages are an attractive therapeutic target because they have a multifaceted cancer-promoting function and are abundant in the tumor microenvironment [4]. We have previously reported that TAM enhances the proliferative and stem cell potential of HCC cells and contributes to tumor progression [5,6].

Programmed cell death protein 1 (PD-1) and its ligand, programmed cell death ligand 1 (PD-L1), are key molecules in the regulation of immune responses [7]. PD-L1 can be expressed by various cells within the TIME, contributing to the formation of an immunosuppressive environment that further promotes tumor progression. In the TIME, TAMs have been identified as major cells expressing PD-L1 [8]. PD-L1(+) TAMs have been reported to possess immunological functions, including the suppression of CD8(+) T cell activity [9,10] and the reduction of activated T cell numbers through apoptosis [11]. However, the immunological functional role of PD-L1–expressing TAMs in HCC has not been fully elucidated.

In this study, the immunological role of PD-L1(+) TAMs in HCC was investigated through single-cell transcriptomic analysis and spatial analysis using multiplex immunohistochemistry (mIHC) staining. The analyses revealed that intratumoral PD-L1(+) TAMs contribute to CD8(+) T cell exhaustion and the formation of the tumor immune microenvironment. These findings suggest the potential for developing novel immunotherapeutic strategies targeting PD-L1(+) TAMs.

## Materials and methods

### Data acquisition and preprocessing

Single-cell RNA sequencing (scRNA-seq) data of hepatocellular carcinoma (HCC) with accession number GSE189903 (GPL24676) [12] were obtained from the Gene Expression Omnibus (GEO). The dataset was generated with the 10x Genomics Chromium single-cell platform. Quality control and preprocessing were performed using the Seurat package (version 5.3.0) in R software. Cells expressing fewer than 200 or more than 5,000 genes, as well as those with a mitochondrial gene proportion exceeding 20%, calculated using the PercentageFeatureSet function for genes starting with “MT-,” were excluded. Genes expressed in three or fewer cells were also removed. Doublets were detected and eliminated using DoubletFinder. Cells annotated with “HCC” in the Histology metadata column and with TissueRegion information indicating AdjacentNormal, TumorBorder, or TumorCore were extracted to generate the HCC subset for subsequent analyses. After quality control, gene expression data were normalized using the NormalizeData function, and the top 2,000 highly variable genes (HVGs) were identified using the FindVariableFeatures function. Data were then scaled with the ScaleData function, regressing out the effects of mitochondrial gene content (percent.mt).

### Dimensionality reduction and clustering

Dimensionality reduction was performed on the normalized and scaled expression data of the HCC subset. Principal component analysis (PCA) was conducted using the RunPCA function, and the top 30 principal components (PCs) were extracted. Based on these PCs, two-dimensional UMAP embeddings were calculated using the RunUMAP function to visualize cellular distributions. For clustering, a nearest-neighbor graph was constructed using the FindNeighbors function on PCs 1 to 30, followed by clustering with the FindClusters function at a resolution of 1.0 using the Louvain algorithm (algorithm = 1). Cells were grouped into clusters based on similarities in gene expression profiles.

### Annotation of Cell clusters

Cell type annotation of the HCC subset was performed by identifying cluster-specific marker genes using the FindMarkers function, selecting positive markers expressed in at least 25% of cells and with a log2 fold-change of 0.25 or greater. The identified markers, including CD8A and CD8B for CD8+ T cells, CD79A and MS4A1 for B cells, CD1C and LAMP3 for dendritic cells, and ALB and APOA2 for hepatocyte-like cells, were compared with information from previous studies and public databases such as CellMarker 2.0 and PanglaoDB. Expression patterns of these markers were visually examined using the FeaturePlot and VlnPlot functions. Based on this information, detailed cell-type annotations (e.g., “CD8+ T cell (Naive/Central Mem),” “Macrophage,” “Hepatocyte-like Cell”) were assigned to each cluster. These annotations were added as new metadata columns in the Seurat object, and UMAP plots displaying annotated clusters were generated. Additionally, detailed cell types were mapped to broader categories (Epithelial, Endothelial, Fibroblasts, CD8+ T cells, Conventional CD4+ T cells, Regulatory T cells, Natural Killer cells, B cells, Monocytes, Macrophages, Dendritic cells, Other cells), which were also stored as metadata and visualized in UMAP plots.

### Differential gene expression and pathway enrichment analyses

To characterize the molecular features of PD-L1(+) macrophages in the TumorCore region of HCC, cells with expression levels of CD45 and CD68 exceeding a threshold of 0.5 were first identified as “CD45(+)CD68(+) cells.” Among these cells, those with a raw count greater than zero for PD-L1 (CD274) were classified as PD-L1(+), while those with a count of zero were classified as PD-L1(−). Differentially expressed genes (DEGs) between PD-L1(+) and PD-L1(−) CD45(+)CD68(+) cells were identified using the FindMarkers function, employing the Wilcoxon rank-sum test. Genes expressed in at least 10% of cells and exhibiting a log2 fold-change of 0.25 or higher were considered. A volcano plot was generated to visualize the DEG results. Furthermore, gene set enrichment analysis (GSEA) was performed based on the DEG results. Genes were ranked by log2 fold-change, and enrichment analyses were conducted using the clusterProfiler package against the KEGG (Kyoto Encyclopedia of Genes and Genomes) pathways and Gene Ontology (GO) databases, including Biological Process (BP), Cellular Component (CC), and Molecular Function (MF). GSEA was conducted using the gseKEGG and gseGO functions, with a significance threshold set at a p-value cutoff of 0.05.

### Analysis of human tissue samples

We analyzed 113 patients with HCC who underwent hepatectomy at the University of Fukui Hospital between April 2006 and January 2024 (Table 1). We evaluated the number of tumors, postoperative recurrence period, survival time, and histological analysis of the resected HCC tissues. Cases that recurred within 5 years following surgery were defined as recurrence cases, and those that did not recur within 5 years were defined as non-recurrence cases. This study was conducted in accordance with the Declaration of Helsinki. The University of Fukui Research Ethics Committee approved the study design (registration number 20210168).

**Table 1:** Characteristics of patients with HCC who underwent hepatectomy.

Age, median (IQR), years	72 (65–76)
Sex, male/female, n	85/28
Etiology, HBV/HCV/NBNC, n	20/39/54
PLT, × 10^9^/L, median (IQR)	156 (122–202)
PT, %, median (IQR)	88.9 (80.8–101.2)
ALT, IU/L, median (IQR)	24 (19–41)
ALB, g/dL, median (IQR)	4.0 (3.7–4.3)
T-bil, g/dL, median (IQR)	0.8 (0.6–1.0)
Child-Pugh score, 5/6/7, n	89/18/6
modified ALBI grade, 1/2a/2b/3, n	69/26/18/0
AFP, ng/mL, median (IQR)	7.1 (3.9–67.3)
DCP, mAU/mL, median (IQR)	50 (25–324)
Maximam tumor size, mm, median (IQR)	28 (20–40)
Tumor multiplicity, single/multiple, n	89/24
Tumor differentiation, well/moderate/poor, n	44/59/10
Vascular invasion	
Vp, 0-1/2-3, n	106/7
Vv, 0-1/2, n	108/5
Va, 0-1/2, n	109/4
BCLC stage, 0/A/B/C, n	21/73/11/8
Fibrosis, 0/1/2/3/4, n	8/21/25/23/36

Abbreviations: AFP, a-fetoprotein; ALB, albumin; ALBI, albumin-bilirubin; ALT, alanine aminotransferase; BCLC stage, Barcelona Clinic Liver Cancer stage; DCP, des-gamma-carboxy prothrombin; HBV, hepatitis B virus; HCV, hepatitis C virus; IQR, interquartile range; NBNC, nonB-nonC; PLT, platelet; PT, prothrombin time; T-bil, total bilirubin.

### Etiology of liver diseases

The etiology of HCC was classified as hepatitis C virus (HCV) and hepatitis B virus for patients testing positive for anti-HCV antibodies (HCV Ab) and hepatitis B surface antigen (HBsAg), respectively. Patients who tested negative for anti-HCV Ab and HBsAg were categorized as non-B, non-C (NBNC).

### Assessment of hepatic reserve function

The albumin–bilirubin (ALBI) score was calculated using the following formula: ALBI score = (log10 bilirubin (µmol/L) × 0.66) + (albumin (g/L) × –0.085). Based on the score, ALBI grades were assigned as follows: ≤ –2.60, Grade 1; > –2.60 to ≤ –1.39, Grade 2; and > –1.39, Grade 3 [13]. Grade 2 was further subdivided into two subcategories, 2a and 2b, using a previously established ALBI score cutoff value of –2.270. These four categories were collectively referred to as modified ALBI grades [14].

### Multiplexed immunofluorescence staining

The Opal Multiplex immunohistochemistry (IHC) Assay Kit (PerkinElmer/Akoya Biosciences) was used according to the manufacturer's protocol. Formalin-fixed paraffin-embedded tumor specimens were cut to 4 µm and dried overnight in air. Then, the tissues were incubated at 65°C for 4 h, and residual paraffin was removed with xylene. The tissues were rehydrated by replacing alcohol with distilled water. Endogenous peroxidase activity was inhibited by treatment with 0.3% hydrogen peroxide (H_2_O_2_). The tissues were boiled using microwave treatment with antigen retrieval (AR) 6 or AR9 buffer for 15 min. Blocking was performed using antibody diluent/block. A panel of primary antibodies were incubated for 30 min. The primary antibody was reacted with Opal Polymer HRP Ms + Rb or goat anti-rat immunoglobin G (IgG) H&L (HRP) preadsorbed (Abcam) or goat anti-rabbit IgG H&L (HRP) preadsorbed (Abcam) for 10 min and visualized for 10 min using Tyramide Signal Amplification (Opal 7-Color IHC, PerkinElmer/Akoya Biosciences). The panel was stained with the following primary antibody/Opal conjugate pair. Human; i) CD8/Opal520, CXCL9/Opal 540, PD-L1/Opal 570, CXCL10/Opal 620, CD163/Opal 650, and CXCR3/Opal 690. ii) CD8/Opal 520, PD-1/Opal 540, PD-L1/Opal 570, TIM3/Opal620, CD163/Opal 650, and CD68/Opal 690. iii) CD3/Opal 520, CD8/Opal 540, PD-L1/Opal 570, CD163/Opal 650, and CD68/Opal 690. Mouse; i) CD3/Opal 520, F4/80/Opal 540, CD163/Opal 570, PD-L1/Opal 620, CD8a/Opal 650, and TIM3/Opal 690. ii) CD3/Opal 520, F4/80/Opal 540, CD163/Opal 570, PD-L1/Opal 620, CD8a/Opal 650, and Granzyme B/Opal 690. iii) CD3/Opal 520, F4/80/Opal 540, CD163/Opal 570, PD-L1/Opal 620, CD8a/Opal 650, and TIM3/Opal 690. During subsequent staining, the tissues were boiled for 15 min in AR buffer at pH 6 or 9 to remove the primary antibody complexes from the samples. For final staining, 4’-6-diamidino-2-phenylindole (DAPI) was added for 10 min and sealed using ProLong Diamond Antifade Mountant (Thermo Fisher Scientific, Waltham, MA, USA) according to the manufacturer's protocol. The slides were imaged at 20 × magnification on the Mantra® Quantitative Pathology Workstation (PerkinElmer/Akoya Biosciences). Color separation, tissue and cell segmentation, and cell phenotyping were performed on inForm® Software v2.6 to extract image data. Cell segmentation was performed on all cells stained with DAPI. Cell phenotyping on inForm® was performed by selecting at least five representative cells per phenotype, performing reiterations as required until at least 20 representative cells per phenotype were selected. Cell classification was performed using the following surface markers: (i) Human; CD3+CD8+ for CD8(+) T cells, CD68+CD163+ for TAMs, (ii) Mouse; CD3+CD8a+ for CD8(+) T cells, F4/80+CD163+ for TAMs. Batch analysis was performed using the same algorithm. phenoptr® (PerkinElmer/Akoya Biosciences) and phenoptrReports® (PerkinElmer/Akoya Biosciences) were used to identify cellular subsets and perform spatial analyses, including the calculation of cell–cell distances. The distance between two cell subtypes was calculated using the *x* and *y* coordinates derived from the inForm® raw data. Per-cell nearest neighbor distances were calculated using phenoptr®. Detailed information on reagents and resources is listed in Supplementary Table S1.

### Interaction variable

**Fig. 1:**
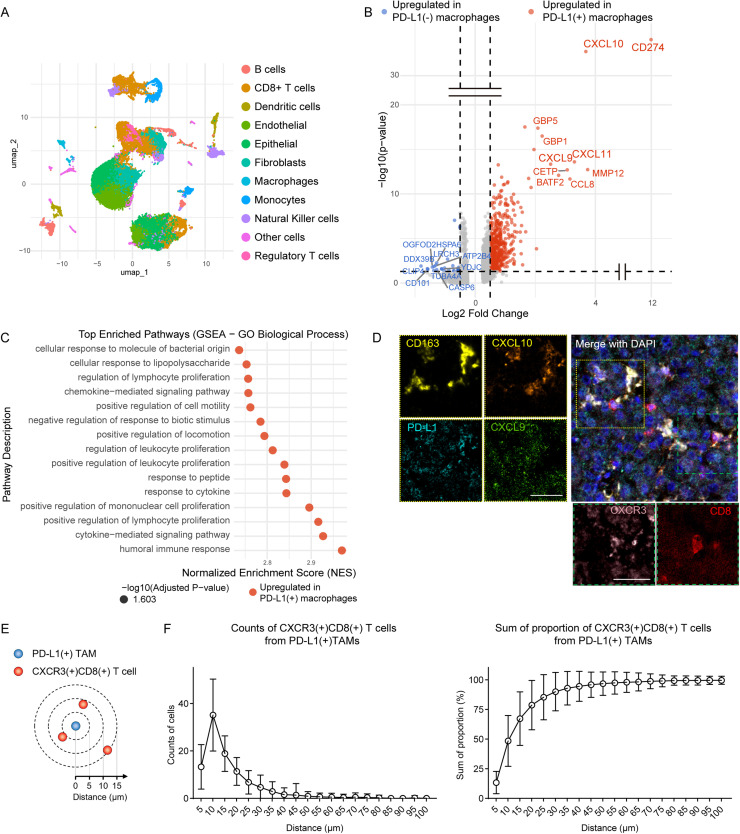
**Single-Cell Transcriptomic Analysis of PD-L1(+) TAMs in hepatocellular carcinoma**. (A) UMAP plot of cell types in resected HCC tissue, colored according to clusters identified by single-cell RNA sequencing analysis (GSE189903). (B) Volcano plot showing differential gene expression between PD-L1(+) and PD-L1(−) macrophages in the tumor region. (C) GO Biological Processes enriched in PD-L1(+) and PD-L1(−) macrophages in the tumor area, identified by GSEA analysis. (D) Multiplex immunofluorescence staining in HCC tissue showing various immune cell markers. (DAPI, blue; CD163, yellow; PD-L1, cyan; CXCL10, orange; CXCL9, green; CD8, red; CXCR3, pink). Bar, 25 μm. (E) Schematic diagram showing the number and distribution of CXCR3(+)CD8(+) T cells measured at 5 µm intervals from PD-L1(+) TAM. (F) Counts (left panel) and sum of proportion (right panel) of CXCR3(+)CD8(+) T cells from PD-L1(+) TAMs. Mean ± SD.

An interaction variable was derived for each pair of immune cell types [15]. Within each image, the total count of CD8(+) T cells that had at least one PD-L1(+) TAM within 25 mm was quantified as the PD-L1(+) TAM with CD8(+) T cells interaction variable. The count was then divided by the total number of CD8(+) T cells and PD-L1(+) TAMs within the area for a given image and multiplied by 100 (Fig. 1D for representation of interaction variable between CD8(+) T cells and PD-L1(+) TAMs) to minimize the effect of CD8(+) T cell or PD-L1(+) TAM presence alone. For Kaplan–Meier curves, patients were classified as having a high interaction sample between two immune cells if they expressed above the median value of the interaction variable and a low interaction sample, if below the median.

### Histological and immunohistochemical analysis

Human and mouse liver tissues were fixed in 10% formaldehyde, embedded in paraffin, and stained with hematoxylin and eosin. In parallel, tissues were stained for immunohistochemistry analysis. For antigen removal, deparaffinized slides were autoclaved in Bond^TM^ Epitope Retrial Solution 1 or 2 (Leica Biosystems, Buffalo Grove, IL, USA) at 121°C for 20 min. Endogenous peroxidase activity was blocked using 0.3% H_2_O_2_ for 15 min, and the slides were incubated with an antibody diluent/block (PerkinElmer/Akoya Biosciences, CA, USA). The sections were further incubated with an optimal dilution of antibodies. The immunoconjugates were detected using Histofine SimpleStain MAX PO (MULTI), Histofine SimpleStain mouse MAX PO (M), or rabbit (R) (Nichirei, Tokyo, Japan) according to the manufacturer's instructions. Samples were imaged at 20 × magnification using a Mantra microscope (PerkinElmer/Akoya Biosciences), and the images were analyzed using inForm® software v2.6 (PerkinElmer/Akoya Biosciences). Positive cells were measured in five randomly chosen visual fields. Detailed information on reagents and resources is listed in Supplementary Table S1.

### Automated histological image analysis

Image analysis was performed using inForm® software v2.6 (Akoya Bioscience). Multiple representative images were selected as batch regions for training. Cell segmentation and phenotyping were performed to assign each cell to a phenotypic category. Histological scores (H-scores) were analyzed using the 4-bin algorithm and calculated based on the PD-L1 staining intensity using this formula: H-score = (1 × % of weakly stained cells) + (2 × % of moderately stained cells) + (3 × % of strongly stained cells).

### Mice

Specific pathogen-free 6- to 7-week-old male BALB/c mice were purchased from Charles River, Japan. Mice were maintained under specific pathogen-free conditions. All animal experiments in this study complied with the University of Fukui’s Regulations for Animal Research (Registration No. R03030).

### Cell lines

The mouse HCC cell line, BNL 1ME A.7R.1 (BNL), was purchased from the American Type Culture Collection and maintained in Dulbecco's modified Eagle medium (DMEM) (Sigma Chemical Co., St Louis, MO, USA) containing 10% fetal bovine serum (FBS), 0.1 mmol/L nonessential amino acids, 1 μmol/L sodium pyruvate, 2 mmol/L l-glutamine, 50 μg/mL streptomycin, and 100 units/mL penicillin (Life Technologies, Inc, Gaithersburg, MD, USA) in a humidified incubator at 37°C in 5% CO_2_. Detailed information on reagents and resources is listed in Supplementary Table S1.

### HCC intrahepatic metastasis mouse model

The BNL cells were collected and resuspended in phosphate-buffered saline (PBS) at a cell density of 2 × 10^5^/ 200 µL. Under anesthesia, this BNL cell suspension (200 µL) was injected into the portal vein of mice using a 32-gauge needle. In some experiments, *InVivo*MAb anti-mouse GM-CSF (Bio X Cell) or *InVivo*MAb rat IgG2a 2a isotype control, anti-trinitrophenol (Bio X Cell) dissolved in PBS was intraperitoneally administered to mice at 200 µg/dose thrice weekly starting from day one. *InVivo*MAb anti-mouse PD-L1 (B7-H1) (Bio X Cell) or *InVivo*MAb rat IgG2b isotype control anti-keyhole limpet hemocyanin (Bio X Cell) dissolved in PBS was intraperitoneally administered at 250 µg/dose twice weekly starting from day one. After 17 or 21 days, the liver was removed and weighed. The weight of liver tumors was determined by measuring the liver weight of mice that received only PBS and subtracting it from the weight of the liver with HCC tumors. Liver tumor tissues were enzymatically dissociated using a Tumor Dissociation Kit (Miltenyi, Bergisch Gladbach, Germany) and a gentleMACS Dissociator (Miltenyi) according to the manufacturer’s protocol. Following dissociation, cells were isolated using CD8 (TIL) MicroBeads (Miltenyi) and Anti-F4/80 MicroBeads UltraPure (Miltenyi), following the manufacturer’s instructions.

### Quantitative gene expression analysis

Total RNA was extracted from cells using an RNeasy Mini Kit (Qiagen, Hilden, Germany). cDNA was synthesized from the RNA using a high-capacity RNA-to-cDNA Kit (Applied Biosystems, Foster City, CA, USA), and quantitative real-time polymerase chain reaction (qRT-PCR) was conducted using the StepOne Plus real-time PCR system (Applied Biosystems). Primers and probes were obtained from Applied Biosystems. The expression levels of target genes were analyzed using the ΔΔCt comparative threshold method. *Glyceraldehyde-3-phosphate dehydrogenase* was used as an internal control.

### Preparation of mouse bone marrow-derived macrophages

Bone marrow cells were extracted from the femurs and tibias in BALB/c mice and cultured in Roswell Park Memorial Institute 1640 medium supplemented with 10% FBS, 1% penicillin/streptomycin, and GM-CSF (40 ng/mL) for 6 days. On day 3, half of the above medium was added to the bone marrow cells. On day 5, unattached cells were discarded, and the above medium was added. On day 7, further experiments were conducted using differentiated Bone Marrow-Derived Macrophages (BMDMs).

### Co-culture of bone marrow-derived macrophages and CD8(+) T cells

Mononuclear suspensions were recovered from mouse spleens and the cells were passed through a 70 μm nylon mesh. Erythrocytes were lysed with ammonium chloride buffer and counted using a hemocytometer [16]. Mouse CD8(+) T cells were isolated using a CD8a+ T Cell Isolation Kit according to the manufacturer’s protocols (Miltenyi). BMDMs were cultured at 3 × 10^5^ cells/well with GM-CSF or PBS for 48 h, washed with PBS, and co-cultured with collected CD8(+) T cells (1.2 × 10^6^ cells/well) for 48h. After washing with PBS, the cells were detached with trypsin and incubated with CD8a+ T Cell Isolation Kit, and CD8(+) T cells were collected for subsequent experiments using magnetic columns.

### Statistical analyses

Statistical significance was determined using the Mann–Whitney U test or one-way analysis of variance followed by the Tukey–Kramer post-hoc test. Cumulative survival was analyzed using the Kaplan–Meier method, and differences were analyzed using the log-rank test. Clinicopathologic factors of survival were identified using the Cox proportional hazards model. Factors that were significant at p < 0.05 in univariate analysis were included in the multivariate analysis. Statistical analyses were performed using Prism software (version 9; GraphPad, GraphPad Software Inc., San Diego, CA, USA). p values < 0.05 were considered statistically significant. Any comparisons not shown on graphs are non-significant.

## Results

### Characteristics of PD-L1(+) tumor-associated macrophages in HCC

To investigate the systematic features of macrophages in HCC progression, the scRNA-seq dataset of Ma et al. (GSE189903) was utilized [12]. The analysis revealed a total of 32 different clusters, each characterized by specific markers, and the cells were subsequently categorized into 10 major cell types (Fig. 1A). When tumor-infiltrating macrophages were classified into PD-L1(+) and PD-L1(–) subsets, the PD-L1(+) macrophages exhibited high expression of the chemokines CXCL9, CXCL10, and CXCL11 (Fig. 1B). Comparison of gene expression profiles between PD-L1(+) TAMs and PD-L1(−) TAMs using GSEA-based GO Biological Process analysis revealed that PD-L1(+) TAMs exhibited significant enrichment of pathways related to immune responses and intercellular communication, including “humoral immune response,” “cytokine-mediated signaling pathway,” and “positive regulation of lymphocyte proliferation” (Fig. 1C). mIHC analysis of resected HCC tissue revealed that CD163(+) macrophages in tumor areas highly expressed PD-L1 as well as CXCL9 and CXCL10 (Fig. 1D). Additionally, CD8(+) T cells expressed the chemokine receptor CXCR3. Spatial distribution analysis centered on PD-L1(+) TAMs revealed that 85.3% of CXCR3(+)CD8(+) T cells were located within a 25 µm radius (Fig. 1E and F). These results suggest that PD-L1(+) TAMs may be involved in modulating immune responses and the immune microenvironment in HCC through the production of chemokines and interactions with immune cells.

### PD-L1(+) Macrophage-induced suppression of CD8(+) T cell anti-tumor responses

**Fig. 2:**
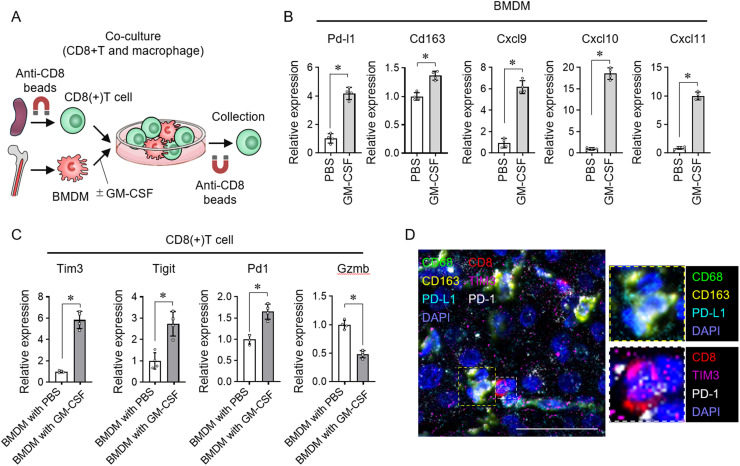
**Co-Culture Experiments to Analyze the Impact of GM-CSF–Induced PD-L1(+) Macrophages on CD8(+) T Cells**. (A) Schematic representation of the experimental procedure. CD8(+) T cells were isolated from the spleens of mice and co-cultured for 48 hours with BMDMs that had been treated with GM-CSF or PBS. Subsequently, the CD8(+) T cells were collected. (B) mRNA expression in BMDMs treated with GM-CSF or PBS was evaluated by qRT-PCR (n = 4). (C) mRNA expression of CD8(+) T cells co-cultured with BMDMs was analyzed by qRT-PCR (n = 4). (D) Multiplex immunofluorescence staining in HCC tissue showing various immune cell markers. (DAPI, blue; CD68, green; CD163, yellow; PD-L1, cyan; CD8, red; TIM3, magenta; PD-1, pink). Bar, 25 μm. (B, C) Mann–Whitney test. * p < 0.05.

PD-L1 and the chemokines CXCL9 and CXCL10 in macrophages have been reported to be induced by the cytokine GM-CSF [17,18]. To enhance the expression of PD-L1 in BMDMs, GM-CSF was added, and the cells were subsequently co-cultured with CD8(+) T cells (Fig. 2A and B). In BMDMs, increased expression of PD-L1 and CD163 was observed along with elevated levels of the chemokines CXCL9, CXCL10, and CXCL11 (Fig. 2B). When CD8(+) T cells were co-cultured with BMDMs highly expressing PD-L1, the expression of TIM3, TIGIT, and PD-1 was increased, whereas the expression of GZMB was decreased (Fig. 2C). In resected HCC tissue, mIHC analysis demonstrated that CD8(+) T cells neighboring PD-L1–positive CD163(+) macrophages within the tumor expressed TIM3 and PD-1 (Fig. 2D). These findings indicate that PD-L1(+) macrophages can suppress the anti-tumor immune responses of CD8(+) T cells.

### Interaction of PD-L1(+) TAMs and CD8(+) T cells in HCC tissue

**Fig. 3:**
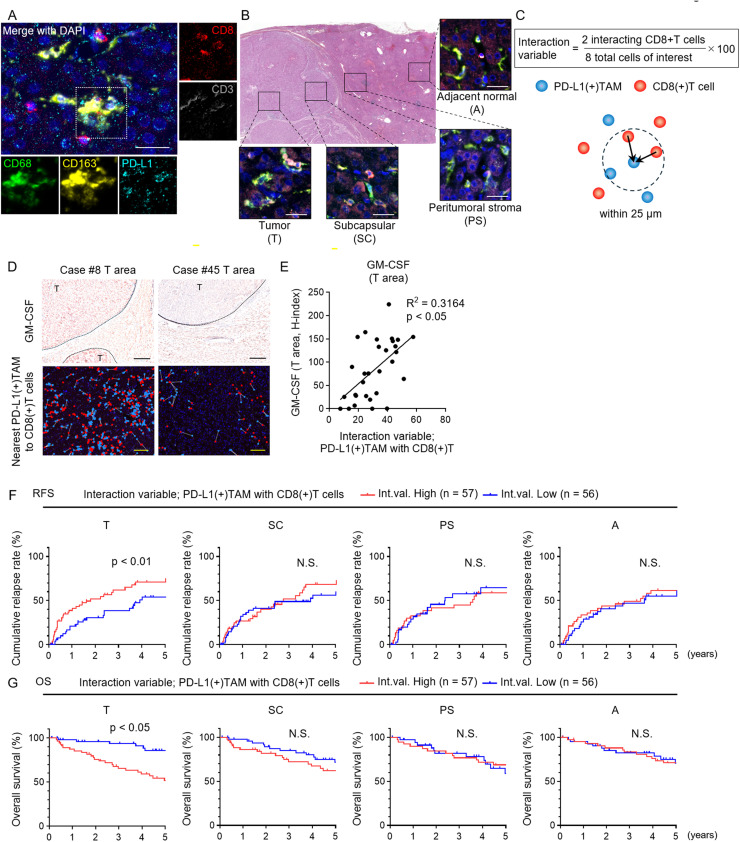
**Spatial analysis of PD-L1(+) tumor-associated macrophage (TAM) and CD8(+) T cell interactions in hepatocellular carcinoma tissue**. (A) Multiplex immunofluorescence staining for immune cell markers in HCC tissue (DAPI, blue; CD8, red; CD3, pink; CD68, green; CD163, yellow; PD-L1, cyan). Bar, 25 μm. (B) HE-stained whole-section image of HCC tissue with areas defined as tumor (T), subcapsular (SC), peritumoral stroma (PS), and adjacent normal (A). Corresponding mIHC images shown. Bar, 5 mm. (C) Schematic of how the interaction variable was calculated based on the proximity (<25 μm) between PD-L1(+) TAMs and CD8(+) T cells. (D) GM-CSF staining in tumor areas (upper) and nearest-neighbor mapping of PD-L1(+) TAMs (blue) and CD8(+) T cells (red) (lower). Bars, upper 500 μm; lower 50 μm. (E) Correlation between GM-CSF H-score and PD-L1(+) TAM–CD8(+) T cell interaction variable in HCC tumors (n = 30). (F, G) Kaplan–Meier curves for relapse-free survival (F) and overall survival (G) in HCC patients after resection, stratified by high or low interaction variable of PD-L1(+) TAMs and CD8(+) T cells (Int.val. High n = 57; Int.val. Low n = 56) in each area. (E) Simple linear regression analysis. (F, G) Log-rank test. * p < 0.05, N.S., not significant.

mIHC was performed on resected specimens from 113 patients with HCC to evaluate the interactions between PD-L1(+) TAMs and CD8(+) T cells based on histological spatial proximity, and to analyze their impact on clinical prognosis (Fig. 3A, Table 1). The evaluation was performed in four areas: the tumor (T), subcapsular (SC), peritumoral stroma (PS), and adjacent normal tissue (A) (Fig. 3B). Spatial proximity was assessed using an interaction variable calculated by counting the number of CD8(+) T cells located within 25 µm of PD-L1(+) TAMs (Fig. 3C). The interaction variable in the T area showed a significant positive correlation with the H-index of GM-CSF (Fig. 3D and E). Among the four areas, patients with a high interaction variable in the T area had significantly worse RFS and OS (Fig. 3F and G). Multivariate analysis revealed that vascular invasion, fibrosis, and the interaction variable between PD-L1(+) TAMs and CD8(+) T cells in the T area were independent factors for postoperative recurrence after HCC resection (Table 2). These results suggest that high spatial proximity between PD-L1(+) TAMs and CD8(+) T cells within the tumor may serve as an independent risk factor for postoperative recurrence and poor prognosis in HCC.

**Table 2:** Univariate and multivariate analysis of clinicopathologic factors associated with survival after hepatocellular carcinoma resection.

		Univariate analysis	Multivariate analysis		
Variables	Patients (n = 113)	p value	Odds ratio	95% CI	p value
Age, y, (≤ 70/ > 70)	45/68	0.845			
Sex, (male/female)	28/85	0.911			
Etiology, (HBV, HCV/NBNC)	59/54	0.849			
PLT, × 10^9^/L, (≤ 150/ > 150)	47/66	0.719			
PT, % (≤ 70/ >70)	11/102	0.421			
ALT, IU/L, (< 30/ ≥ 30)	68/45	0.708			
ALB, g/dL, (≤ 3.5/ > 3.5)	15/98	0.542			
T-bil, mg/dL, (< 1.1/ ≥ 1.1)	88/25	0.079			
Child-Pugh score, (5/ ≥ 6)	89/24	0.357			
mALBI grade, (1,2a/2b,3)	69/44	0.347			
AFP, ng/mL, (< 13.4/ ≥ 13.4)	70/43	**0.031**	0.727	0.428–1.244	0.240
DCP, mAU/mL, (< 40/ ≥ 40)	42/71	0.807			
Maximam tumor size, mm, (< 30/ ≥ 30)	60/53	0.044			
Tumor multiplicity, (single/multiple)	89/24	0.514			
Tumor differentiation, (well/moderate, poor)	44/69	0.143			
Vascular invasion, (absent/present)	76/37	**0.022**	0.569	0.335–0.983	**0.039**
BCLC stage (0,A/B,C)	94/19	0.175			
Fibrosis, (F0-3/F4)	77/36	**0.043**	0.563	0.323–0.993	**0.045**
Counts of PD-L1(+)TAMs, T area (< median/ ≥ median)	56/57	0.315			
Counts of CD8(+)T cells, T area (< median/ ≥ median)	56/57	0.644			
Interaction variable; PD-L1(+)TAM with CD8(+)T cells (< median/ ≥ median)	56/57	**0.018**	0.557	0.328–0.928	**0.026**

Abbreviations: AFP, a-fetoprotein; ALB, albumin; ALBI, albumin-bilirubin; ALT, alanine aminotransferase; BCLC stage, Barcelona Clinic Liver Cancer stage; DCP, des-gamma-carboxy prothrombin; PLT, platelet; PT, prothrombin time; T-bil, total bilirubin.

### Enhancement of anti-tumor immunity via inhibition of PD-L1(+) TAMs using anti-GM-CSF and anti-PD-L1 antibodies

**Fig. 4:**
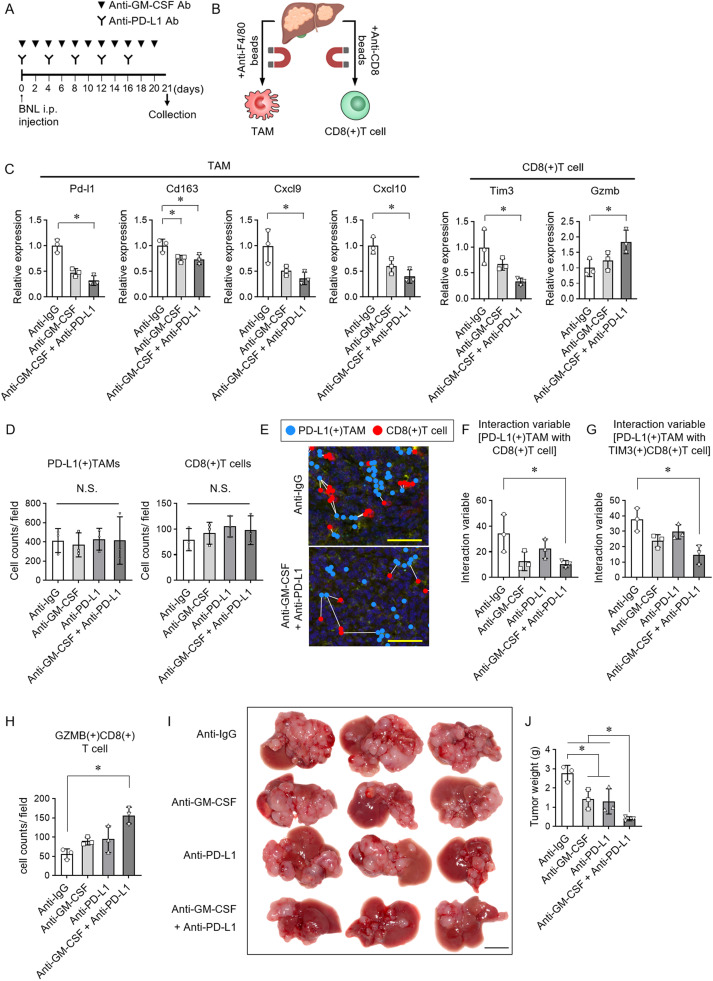
**Anti-GM-CSF and anti-PD-L1 antibodies enhance anti-tumor immunity by inhibiting TAM-CD8(+) T cell interactions**. (A) Experimental design. Mice were injected with BNL cells via the portal vein and subsequently administered anti-GM-CSF and anti-PD-L1 antibodies or isotype IgG intraperitoneally according to the schedule shown. Tissue samples were collected on day 21. (B) Liver tumors were dissociated into single cells, and F4/80(+) macrophages and CD8(+) T cells were purified using magnetic bead separation. (C) Gene expression levels in tumor-associated macrophages (TAMs) and CD8(+) T cells from liver tumors were analyzed by qRT-PCR. (D) The number of PD-L1(+) TAMs and CD8(+) T cells per field in the tumor. (E) Nearest neighbor diagrams showing spatial relationships between PD-L1(+) TAMs (blue) and CD8(+) T cells (red) in liver tumors. Scale bar, 25 μm. (F, G) Interaction variable of (F) PD-L1(+) TAM with CD8(+) T cell and (G) PD-L1(+) TAM with TIM3(+)CD8(+) T cell in tumor area. (H) Cell counts of GZMB(+)CD8(+) T cells in the tumor area of the liver tissue. (I) Representative macroscopic images of liver tumors from mice treated with anti-IgG, anti-GM-CSF antibody, anti-PD-L1 antibody, or their combination. Scale bar, 10 mm. (J) Tumor weights in each treatment group. (C, D, E-H, J) Tukey–Kramer post hoc test. * p < 0.05.

In a mouse liver tumor model, anti-GM-CSF and anti-PD-L1 antibodies were administered, and tissues were harvested 21 days later (Fig. 4A). TAMs and CD8(+) T cells were separated from the tumors for RNA expression analysis (Fig. 4B). Treatment with these antibodies reduced PD-L1, CD163, CXCL9, CXCL10, and CXCL11 expression in TAMs and led to decreased TIM3 and increased GZMB expression in CD8(+) T cells (Fig. 4C). The numbers of intratumoral PD-L1(+) TAMs and CD8(+) T cells were unaffected by administration of anti-GM-CSF and anti-PD-L1 antibodies (Fig. 4D). Combined administration of anti-GM-CSF and anti-PD-L1 antibodies significantly reduced the interaction variable between PD-L1(+) TAMs and CD8(+) T cells within the tumor (Fig. 4E and F). The interaction between PD-L1(+) TAMs and TIM3-expressing CD8(+) T cells was also lower in the combination group (Fig. 4G), while the number of CD8(+) T cells expressing granzyme B increased (Fig. 4H). Both anti-GM-CSF and anti-PD-L1 antibodies significantly inhibited tumor growth compared to the anti-IgG control group, and their combination further enhanced the anti-tumor effect (Fig. 4I and J). These results suggest that the inhibition of PD-L1(+) TAMs by anti-GM-CSF antibody may have synergistically contributed to the tumor-suppressive effect when combined with anti-PD-L1 antibody.

## Discussion

In this study, the immunological functions of PD-L1(+) TAMs in patients with HCC were investigated through single-cell transcriptomic analysis and spatial interaction analysis using mIHC staining. Intratumoral PD-L1(+) TAMs were suggested to produce chemokines and potentially induce exhaustion of CD8(+) T cells. A high spatial proximity between PD-L1(+) TAMs and CD8(+) T cells within tumors was shown to be an independent risk factor for postoperative recurrence and poor prognosis in HCC. The combination of anti-GM-CSF and anti-PD-L1 antibodies was found to suppress the differentiation of PD-L1(+) TAMs and their interactions with CD8(+) T cells, contributing to the alleviation of CD8(+) T cell exhaustion and the enhancement of anti-tumor effects.

TAMs are pivotal in cancer progression, influencing tumor growth, angiogenesis, and immune evasion [19]. TAMs are the main cells expressing PD-L1 in human TIME in HCC [20], gastric cancer [21], ovarian cancer [22], and lung adenocarcinoma [23]. The expression of PD-L1 in TAMs has been suggested to contribute to TAM activation and M2 polarization [24]. PD-L1(+) TAMs have been shown to express M2-related signatures [25], produce immunosuppressive cytokines such as transforming growth factor-β and IL-10 [25], induce suppression of CD8(+) T cell activity [9], and reduce the number of activated T cells via apoptosis [11]. Furthermore, in breast cancer, GM-CSF has been shown to enhance PD-L1 expression on TAMs and inhibit T cell function through activation of the STAT3 pathway [10]. These findings support the notion that intratumoral PD-L1(+) TAMs in the present study may induce exhaustion of CD8(+) T cells. The PD-1/PD-L1 axis is already well established as a key immune checkpoint pathway in cancer [26]. In this study, by combining large-scale multi-regional spatial analysis of 113 resected HCC specimens with functional validation using therapeutic antibody treatment in mouse models, we provide the comprehensive evidence that PD-L1 expressed by TAMs is the driver of CD8(+) T cell exhaustion in HCC. These findings clarify the immunosuppressive role of intratumoral PD-L1(+) TAMs and support the rationale for TAM-targeted strategies to enhance the efficacy of ICI therapy, underscoring the originality and novelty of this work.

In this study, spatial interaction analysis of PD-L1(+) TAMs and CD8(+) T cells using mIHC revealed that their proximity within the tumor leads to immune exhaustion and is an important factor in recurrence and prognosis. Advances in spatial pathological imaging techniques have revealed cell-cell communication networks and niches within tumors by identifying neighboring cell interaction patterns from spatial data [27,28]. The importance of immune cell interactions in tumor progression has also been indicated in patients with HCC [27,29,30]. In residual HCC after transarterial chemoembolization, PD-L1(+) M2-like macrophages interact with stem cell-like tumor cells and correlate with the exhaustion of CD8(+) T cells, using single-cell high multiplex cytometry imaging [31].

GM-CSF induces CXCL9 and CXCL10 in macrophages [17] and activates JAK2/STAT3 signaling, raising PD-L1 levels and suppressing T cell anti-tumor activity, which promotes gastric [18] and hepatocellular cancer [32]. Blocking GM-CSF reduces TAM infiltration and boosts T cell responses, helping inhibit tumor growth [23]. These reports are consistent with the known role of GM-CSF in promoting the expression and differentiation of PD-L1, CXCL9, CXCL10, and CXCL11 in macrophages, as observed in this study.

Although CD8(+) T cells in this study were freshly isolated without cryopreservation, low-level activation was observed under control conditions. This may be explained by weak antigen presentation from BMDMs [33], transient activation during magnetic bead isolation [34], and tonic TCR signaling through self-peptide–MHC interactions [35]. These mechanisms likely account for the basal expression of activation markers in the absence of external stimuli. However, in our experiments, the upregulation of TIM3, TIGIT, and PD1, as well as the downregulation of GZMB, was consistently more pronounced under co-culture with PD-L1–high BMDMs, indicating that these changes extend beyond basal background activation and are dependent on PD-L1(+) macrophages.

This study has several limitations. First, the number of cases analyzed was limited, and caution is required when generalizing the results. Second, although the data suggest a potential causal relationship between PD-L1(+) TAMs and the induction of CD8(+) T cell exhaustion, direct functional experiments to confirm this have not yet been conducted. Third, although spatial analysis was performed using mIHC, further validation through more advanced spatial omics approaches, such as proximity-based ligand–receptor analyses, is warranted. Moreover, caution should be exercised in directly extrapolating the results obtained from the mouse models used in this study to human HCC. Future studies are needed to validate these findings in clinical samples and to assess the efficacy of therapeutic strategies targeting PD-L1(+) TAMs. This study has limitations in protein-level validation of CD8(+) T cell exhaustion markers. Although TIM-3 and PD-1 expressions were confirmed by mIHC in human tumor tissues and TIM-3 expression in mouse tumor tissues, comprehensive FACS analysis in the HCC mouse model and co-culture experiments was not performed. Future studies incorporating FACS will be necessary to quantitatively validate immune marker expression. Furthermore, in this study, tumor burden was evaluated by tumor weight, whereas *in vivo* bioluminescence imaging using luciferase-expressing stable HCC cell lines was not performed. This approach would allow for quantitative and dynamic assessment of tumor burden and should be considered for incorporation in future studies. While this study demonstrates that PD-L1(+) TAMs contribute to CD8(+) T cell exhaustion, direct experimental validation remains necessary. In particular, an *in vitro* cytotoxicity assay using OT-I transgenic mice would provide definitive evidence for antigen-specific CD8(+) T cell functionality suppressed by PD-L1(+) macrophages. Furthermore, the animal experiments were conducted with a small sample size, which may limit the statistical reliability of the findings. The results obtained are consistent across multiple analytical platforms, including scRNA-seq, mIHC, and co-culture systems, thereby supporting the validity of our conclusions. Future studies with additional validation will provide more robust evidence to further substantiate these conclusions.

Collectively, the findings suggest that intratumoral PD-L1(+) TAMs in HCC have the potential to drive CD8(+) T cell exhaustion and modulate the immune microenvironment. Furthermore, the spatial proximity between PD-L1(+) TAMs and CD8(+) T cells was associated with poor prognosis. These results highlight the potential of targeting PD-L1(+) TAMs in developing new immunotherapeutic strategies.

## Funding sources

This research was partially supported by 10.13039/100009619Japan Agency for Medical Research and Development (AMED) under Grant Numbers JP25fk0310529 and JP25fk0210174 and 10.13039/501100001691Japan Society for the Promotion of Science (JSPS) KAKENHI Grant-in-Aid for Scientific Research Number JP25K11170.

## CRediT authorship contribution statement

**Takuto Nosaka:** Writing – review & editing, Writing – original draft, Visualization, Validation, Resources, Project administration, Methodology, Investigation, Funding acquisition, Formal analysis, Data curation. **Masahiro Ohtani:** Resources, Investigation. **Junki Yamashita:** Resources, Investigation. **Yosuke Murata:** Resources, Investigation. **Yu Akazawa:** Resources, Investigation. **Tomoko Tanaka:** Resources, Investigation. **Kazuto Takahashi:** Resources, Investigation. **Tatsushi Naito:** Resources, Investigation. **Yoshiaki Imamura:** Validation, Resources, Investigation. **Kenji Koneri:** Validation, Resources, Investigation. **Takanori Goi:** Validation, Resources, Investigation. **Yasunari Nakamoto:** Writing – review & editing, Writing – original draft, Visualization, Validation, Supervision, Project administration, Methodology, Investigation, Funding acquisition, Data curation, Conceptualization.

## Declaration of competing interest

The authors declare that they have no known competing financial interests or personal relationships that could have appeared to influence the work reported in this paper.

## Appendix Supplementary materials

Image, application 1
