# Supplementary material for: PD-L1(+) tumor-associated macrophages induce CD8(+) T Cell exhaustion in hepatocellular carcinoma
Source: Neoplasia. 2025 Sep 29;69:101234. doi: 10.1016/j.neo.2025.101234 (PMC12513114; doi:10.1016/j.neo.2025.101234)
Supplement: Supplementary file 1 [file mmc1.docx]

**Supplementary Table S3**. Lists of reagents and resources

| REAGENT or RESOURCE | SOURCE | IDENTIFIER |
| --- | --- | --- |
| Antibodies |  |  |
| anti-human CD68 (clone KP1) | Gene Tex | Cat# GTX73643; RRID: AB_375045 |
| anti-human, mouse CD163 (clone EPR19518) | abcam | Cat# ab182422; RRID: AB_2753196 |
| anti-human CD8 (clone 1G2B10) | Proteintech | Cat# 66868-1-Ig; RRID: AB_2882205 |
| anti-human, mouse CD3 (clone SP7) | abcam | Cat# ab16669; RRID: AB_443425 |
| anti-human, mouse PD-L1 [SP142] (clone SP142) | abcam | Cat# ab228462; RRID: AB_2827816 |
| anti-human GM-CSF (clone N/A) | Proteintech | Cat# 17762-1-AP; RRID: AB_2276718 |
| anti-human pan Cytokeratin [AE1/AE3 + 5D3] (clone N/A) | abcam | Cat# ab86734; RRID: AB_10674321 |
| anti-human CD66b (clone N/A) | abcam | Cat# ab197678; RRID: AB_3644234 |
| anti-mouse CD8 alpha (clone EPR21769) | abcam | Cat# ab217344; RRID: AB_2890649 |
| anti-human, mouse TIM3 (clone EPR22241) | abcam | Cat# ab241332; RRID: AB_2888936 |
| anti-human, mouse Granzyme B (clone EPR22645-206) | abcam | Cat# ab255598; RRID: AB_2860567 |
| anti-human PD-1 (clone NAT105) | abcam | Cat# ab52587; RRID: AB_881954 |
| anti-mouse F4/80 (clone Cl:A3-1) | BIO-RAD | Cat# MCA497GA; RRID: AB_323806 |
| Goat Anti-Rat IgG H&L (HRP) preadsorbed | abcam | Cat# ab7097; RRID: AB_955411 |
| Goat Anti-Rabbit IgG H&L (HRP) preadsorbed | abcam | Cat# ab7090; RRID: AB_955417 |
| Goat Anti-Rabbit IgG H&L (Alexa Fluor® 488) | abcam | Cat# ab150077; RRID: AB_2630356 |
| Chemicals, peptides, and recombinant proteins |  |  |
| ProLong™ Diamond Antifade Mountant | Thermo Fisher Scientific | P36961 |
| Bond TM Epitope Retrieval 1 | Leica Biosystems | AR9961 |
| Bond TM Epitope Retrieval 2 | Leica Biosystems | AR9640 |
| Histofine SimpleStain MAX PO (MULTI) | Nichirei | 424151 |
| Histofine SimpleStain mouse MAX PO (M) | Nichirei | 414131 |
| Histofine SimpleStain mouse MAX PO (R) | Nichirei | 414341 |
| Recombinant murine GM-CSF | PeproTech | 315-03 |
| *InVivo*MAb anti-mouse GM-CSF (clone MP1-22E9) | Bio X Cell | BE0259 |
| *InVivo*MAb rat IgG2a isotype control, anti-trinitrophenol (clone 2A3) | Bio X Cell | BE0089 |
| *InVivo*MAb anti-mouse PD-L1 (B7-H1) (clone 10F.9G2™) | Bio X Cell | BE0101 |
| *InVivo*MAb rat IgG2b isotype control, anti-keyhole limpet hemocyanin (clone LTF-2) | Bio X Cell | BE0090 |
| DharmaFECT 4 Transfection Reagent | Horizon Discovery | T-2004 |
| Dulbecco's modified Eagle medium (DMEM) | Thermo Fisher Scientific (Gibco) | 11965092 |
| Nonessential amino acids | Thermo Fisher Scientific (Gibco) | 11140050 |
| Sodium pyruvate | Thermo Fisher Scientific (Gibco) | 11360070 |
| HEPES Buffer Solution | Thermo Fisher Scientific (Gibco) | 15630080 |
| L-glutamine | Thermo Fisher Scientific (Gibco) | 25030081 |
| Penicillin-Streptomycin | Thermo Fisher Scientific (Gibco) | 15140122 |
| Critical commercial assays |  |  |
| Opal 6-Plex Manual Detection Kit | Akoya Biosciences | NEL811001KT |
| Anti-F4/80 MicroBeads UltraPure, mouse | Miltenyi Biotec | 130-110-443 |
| Tumor Dissociation Kit, mouse | Miltenyi Biotec | 130-096-730 |
| CD8 (TIL) MicroBeads, mouse | Miltenyi Biotec | 130-116-478 |
| CD8a+ T Cell Isolation Kit, mouse | Miltenyi Biotec | 130-104-075 |
| RNeasy Mini Kit | Qiagen | 74104 |
| High Capacity RNA-to-cDNA Kit | Applied Biosystems | 4387406 |
| Experimental models: Cell lines |  |  |
| Mouse: BNL 1ME A.7R.1 | American Type Culture Collection | TIB-75 |
| Experimental models: Organisms/strains |  |  |
| Mouse: BALB/cJ | The Jackson Laboratory | Stock # 000651 |
| Oligonucleotides |  |  |
| Taqman probe mouse Cxcl9 | Thermo Fisher Scientific | Mm00434946_m1 |
| Taqman probe mouse Cxcl10 | Thermo Fisher Scientific | Mm00445235_m1 |
| Taqman probe mouse Cxcl11 | Thermo Fisher Scientific | Mm00444662_m1 |
| Taqman probe mouse Cd274 | Thermo Fisher Scientific | Mm03048248_m1 |
| Taqman probe mouse Cd163 | Thermo Fisher Scientific | Mm00474091_m1 |
| Taqman probe mouse Tigit | Thermo Fisher Scientific | Mm03807522_m1 |
| Taqman probe mouse Cd274 | Thermo Fisher Scientific | Mm03048248_m1 |
| Taqman probe mouse Pdcd1 | Thermo Fisher Scientific | Mm01285676_m1 |
| Taqman probe mouse Gzmb | Thermo Fisher Scientific | Mm00442837_m1 |
| Taqman probe mouse Gapdh | Thermo Fisher Scientific | Mm99999915_g1 |
| Software and algorithms |  |  |
| R: The Project for Statistical Computing | N/A | https://www.r-project.org/ |
| RStudio | N/A | https://posit.co/download/rstudio-desktop/ |
| inForm | Akoya Biosciences | https://www.akoyabio.com/phenoimager/inform-tissue-finder/ |
| phenoptrReports & phenoptr | Akoya Biosciences | https://www.akoyabio.com/phenoimager/phenoptrreports-phenoptr/ |
| Prism | GraphPad Software | https://www.graphpad.com/ |
